# Carbon ion radiotherapy in a hypofractionation regimen for stage I non-small-cell lung cancer

**DOI:** 10.1093/jrr/rrt216

**Published:** 2014-03

**Authors:** Wataru Takahashi, Mio Nakajima, Naoyoshi Yamamoto, Hiroshi Tsuji, Tadashi Kamada, Hirohiko Tsujii

**Affiliations:** Research Center of Charged Particle Therapy, National Institute of Radiological Sciences (NIRS), Chiba, Japan

## Abstract

Introduction: In 1994, we started carbon-ion radiotherapy (CIRT) for peripheral stage I non-small-cell lung cancer (NSCLC). First, two phase I/II clinical trials demonstrated the optimal doses of 90.0 GyE in 18 fractions over 6 weeks (Protocol 9303) and 72.0 GyE in 9 fractions over 3 weeks (Protocol 9701) for achieving more than 95% local control with minimal pulmonary toxicity. As a next step, we conducted two successive phase II trials. The first trial (Protocol 9802) used a regimen of 72 GyE per 9 fractions over 3 weeks and the second trial (Protocol 0001) used a regimen of 4 fractions over 1 week, at a fixed dose of 52.8 GyE for stage IA and 60 GyE for IB. In these Phase II trials, the local control rate (LCR) for all patients was 91.5%, and those for T1 and T2 tumors were 96.3 and 84.7%, respectively. The 5-year cause-specific survival rate (CSS) was 67.0% (IA: 84.4, IB: 43.7), and overall survival (OS) was 45.3% (IA: 53.9, IB: 34.2). No adverse events greater than grade 2 occurred in the lung.

In 2003, we also started a phase I/II clinical trial (Protocol 0201) as a dose escalation study using single fraction. The initial total dose was 28.0 GyE administered and escalated in increments of 2.0 GyE each, up to 50.0 GyE. This clinical trial ended in February 2012 and is still followed up. In this article, we investigated the preliminary results of this phase I/II trial.

Materials and methods: In this prospective study, 151 primary stage I NSCLC were treated by CIRT monotherapy using a total dose of 36.0 GyE (*n* = 18), 38.0 GyE (*n* = 14), 40.0 GyE (*n* = 20), 42.0 GyE (*n* = 15), 44.0 GyE (*n* = 44), 46.0 GyE (*n* = 20), 48.0 GyE (*n* = 10) and 50.0 GyE (*n* = 10) using single fractionation. Mean age was 73.9 years, and size of tumor included T1 (*n* = 91) and T2 (*n* = 60). By type (cancer type was determined by biopsy), there were 104 adenocarcinomas, 46 squamous cell carcinomas and 1 large cell carcinoma. Medical inoperability was 55.6%.

The patient is fixed on the rotational couch by using a custom-made immobilization device. Under free breathing conditions, planning CT images were acquired for treatment planning. The clinical target volume (CTV) was determined by adding >10-mm margin to the gross tumor volume (GTV). The planning target volume (PTV) was created by adding an internal margin to CTV as 5 mm in craniocaudal direction. The prescribed dose was delivered to PTV with different coplanar four beam angles. A respiratory-gated irradiation system was used in all irradiation sessions.

Results: The median follow-up time was 45.6 months (range, 1.6–88.4 months). For 151 patients, the 5-year overall LCR was 79.2%, and those for T1 (*n* = 91) and T2 (*n* = 60) tumors were 83.6 and 72.2%, respectively. Also, local control in T1a, T1b, T2a and T2b were 96.8, 84.4, 80.2 and 20.0%, respectively. The OS was 55.1% and the CSS was 73.1%. No toxicity greater than grade 2 was observed in the lung and the skin.

Conclusions: In patients with stage I NSCLC, CIRT using single fraction is considered as a promising curative modality. Especially for elderly and inoperable cases, CIRT could be a minimally invasive therapeutic option as a valid alternative to surgical resection (Fig. 1).

**Fig 1.**
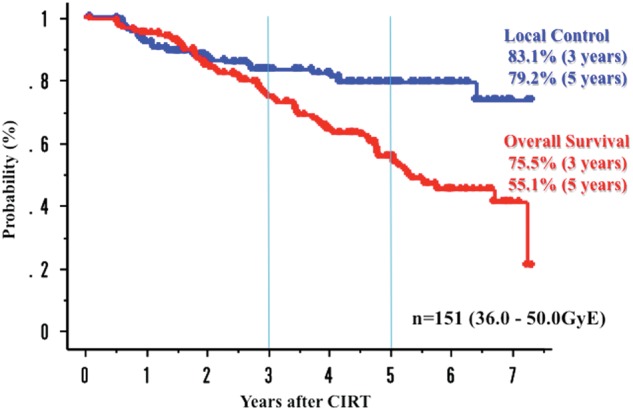
Overall survival and local control in 151 patients with stage I non-small-cell lung cancer.

Overall survival and local control in 151 patients with stage I non-small-cell lung cancer.
